# Trends in multidrug-resistant tuberculosis in Tehran, Iran: an analysis of published data

**DOI:** 10.3205/dgkh000327

**Published:** 2019-08-16

**Authors:** Mohammad Javad Nasiri, Mohsen Heidary, Hossein Goudarzi, Payam Tabarsi

**Affiliations:** 1 Department of Microbiology, School of Medicine, Shahid Beheshti University of Medical Sciences, Tehran, Iran; 2 Student Research Committee, School of Medicine, Iran University of Medical sciences, Tehran, Iran; 3 Department of Microbiology, School of Medicine, Iran University of Medical Sciences, Tehran, Iran; 4 Clinical TB and Epidemiology Research Center, National Research Institute of Tuberculosis and Lung Diseases, Shahid Beheshti University of Medical Sciences, Tehran, Iran

**Keywords:** tuberculosis, MDR-TB, Tehran, Iran

## Abstract

**Introduction:** Multidrug-resistant tuberculosis (MDR-TB) is one of the major public health threats especially in developing countries. In Iran, the emergence and spread of MDR-TB are likely to pose a significant problem for the National Tubeculosis Control Program (NTP). In this study, to determine the trend of MDR-TB in Tehran, the results of published studies were analyzed.

**Methods:** Several databases were searched, including Medline, Embase, Web of Science and Iranian databases. Studies which report the prevalence of MDR-TB by World Health Organization (WHO)-endorsed drug susceptibility testing (DST) methods were included in the study. Data were analyzed with SPSS 20 (SPSS, Chicago, Illinois, USA).

**Results:** Analysis of the MDR-TB trend did not show any increase among new TB cases, but the trend of MDR-TB was significantly increased among previously treated cases. The prevalence of MDR-TB from 48.0% in 1996 reached 58.0% in 2004 (P<0.05).

**Conclusions:** Our analysis shows an increasing trend in MDR-TB, particularly in retreatment cases. This study strongly highlights the need to develop further strategies for surveillance, monitoring, and management of MDR-TB cases.

## Zusammenfassung

**Einleitung:** Die multiresistente Tuberkulose (MDR-TB) ist insbesondere in Entwicklungsländern eine der größten Bedrohungen für die öffentliche Gesundheit. Im Iran stellt die Verbreitung von MDR-TB ein erhebliches Problem für das nationale Tuberkulose-Kontrollprogramm (NTP) dar. Daher sollte der Trend der MDR-TB durch das Nationale Tubekulose-Referenzzentrum im Iran ermittelt werden.

**Methode:** Systematisch wurden in Medline, Embase, Web of Science und iranischen Datenbanken ermittelte Veröffentlichungen des Zeitraums 1996 bis 2017 ausgewertet. Dabei wurden nur Berichte zur Prävalenz von MDR-TB eingeschlossen, sofern die klinische Diagnose durch eine valide Labordiagnose bestätigt worden war. Die Daten wurden mittels SPSS 20 analysiert; eine Irrtumswahrscheinlichkeit von p<0,05 wurde im der Chi-Quadrat-Test als Signifikanzniveau definiert.

**Ergebnisse:** Die Analyse ergab keinen signifikanten Anstieg neuer Fälle von MDR-TB. Dagegen war die Häufigkeit einer Wiederbehandlung von MDR-TB von 1996 mit 48,0% auf 58,0% im Jahr 2004 signifikant angestiegen (p<0,05).

**Schlussfolgerung:** Basierend auf der Analyse ist bei MDR-TB ein zunehmender Trend zu verzeichnen, insbesondere für die Notwendigkeit der Wiederbehandlung. Die Studie unterstreicht die Notwendigkeit der Surveillance und Entwicklung neuer Behandlungsstrategien von MDR-TB.

## Introduction

Multidrug-resistant tuberculosis (MDR-TB) poses serious threats to global TB control programs and remains a major public health concern in many countries. The emergence and spread of MDR-TB, defined as *Mycobacterium tuberculosis* bacilli resistance to isoniazid and rifampicin, is mainly related to poor patient management, inappropriate treatment regimens, as well as the quantity of antibiotics and how they are being used [1], [2]. Treatment for MDR-TB is longer, and requires more expensive, more toxic drugs [3], [4]. For most patients with MDR-TB, the current World Health Organization (WHO)-recommended regimen last 20 months, and treatment success rates are much lower compared to treatment of non-MDR-TB [5]. Identifying the trends in drug resistance is one of the important aspects in the assessment of any TB control program and has significant implications for public health policy [6]. In Iran, several regional hospitals do not have proper facilities for admission of TB patients. Consequently, TB cases have to come to the national TB center in Tehran, the capital of Iran, for further treatment and hospitalization [7]. Thus, in Tehran, TB is one of the major public health problems, exacerbated by the emergence and spread of drug resistant TB [8].

Despite existing reports on drug-resistant TB in Tehran, little is known about the trend of MDR-TB, which could be useful for better management of TB. Thus, to determine the trend of MDR-TB in Tehran, the results of published studies were analyzed.

## Methods

### Search strategy and inclusion criteria

We searched Medline (via PubMed), Embase, Web of Science and Iranian databases for relevant studies published between Jan 1, 1996 and Nov 30, 2017, with the search terms “tuberculosis”, “multidrug-resistance”, “trends”, and related terms. 

Original articles were obtained and assessed in detail for inclusion. Studies had to meet all of the following criteria for inclusion:

1. the data should be representative of TB cases in Tehran or the geographical setting under study;
2. drug resistance among new TB cases was examined separately from drug resistance among previously treated TB cases; and
3. drug susceptibility testing (DST) methods were selected from among those endorsed by the WHO.

Studies that did not met the inclusion criteria were excluded from further analysis.

### Data extraction

 The following information was extracted for each study when provided: first author, year of publication, study setting, distribution of age and sex in the study population, number of patients investigated, prevalence of MDR-TB. 

### Statistical analysis

The statistical significance of observed trends of MDR-TB was tested using the Chi-squared test. All analyses were performed using the statistical software packages SPSS version 20 (SPSS, Chicago, Illinois). An error probability <0.05 was defined as significant.

## Results

Based on the inclusion criteria, 4 original articles were included (Table 1 (Tab. 1)). These studies were conducted in the National Research Institute of Tuberculosis and Lung Diseases (NRITLD) of Iran. This center, located in Tehran, acts as the reference center for the National Tuberculosis Programs (NTP). 

As shown in Table 2 (Tab. 2), the rate of MDR-TB as well as the resistance only against isoniazid (INH) and rifampicin (RIF) in new cases did not show an increase. However, the rate of MDR-TB in previously treated cases showed an increasing trend over the study period from 48.0% in 1996 to 58.0% in 2004 (P<0.05) (Table 3 (Tab. 3)).

## Discussion

To the best of our knowledge, this is the first study in which trends of MDR-TB in the capital of Iran have been investigated over the past years. Overall, the trend of MDR-TB in new cases did not show any increase in the last decade. However, we found a trend toward increasing prevalence of MDR-TB from 1996 (48.0%) to 2004 (58.0%) in previously treated cases.

Although Iran has made enormous progress in successful treatment of drug-susceptible TB in recent years, relatively high rates of drug-resistant TB still exist. According to the latest report released by the WHO in 2016, a total of 10,399 TB cases were documented in Iran, including 130 MDR-TB cases [9]. Based on this report, the prevalence of MDR was generally much higher in previously treated cases than in patients who had not yet received treatment [9]. MDR-TB can be acquired through mutation during previous ineffective anti-TB treatment or through transmission from other MDR-TB cases [10]. However, due to a paucity of molecular epidemiological studies in Iran, it is not known whether MDR-TB can arise from previous ineffective treatment. If it does, then improved treatment of susceptible strains could prevent additional emergence of MDR cases [10].

Currently, the duration of treatment and the choice of drugs for patients with MDR-TB is standardized in Iran [11]. However, this might not be the optimal approach, since the duration of therapy to achieve a relapse-free cure may be quite variable and depend on different factors (e.g., the virulence of the bacilli, the degree of drug resistance, the immune status of the host, drug exposure and tolerability) [12]. According to the National Tuberculosis Control Program (NTP), patients with MDR-TB should be treated with a combination of first- and second-line drugs for a minimum of 20 months [11]. In reality, the recommended lengthy (20 months) treatment might be either excessive or insufficient for the majority of patients. Furthermore, these long-term therapy regimens incur very high costs, due to drug-related expenses and prolonged hospitalization. Recently, standardized drug regimens given for 9–12 months have been safely used to considerably shorten the duration of MDR-TB treatment in some settings [13], [14], [15]. 

Despite the amount of resources dedicated to drug-resistant TB treatment in Iran, the trend of increasing MDR-TB only in re-treatment cases as found by the current study indicates a relatively inefficient drug management system. In addition, in most parts of Iran, due to inadequate laboratory capacity, most of the MDR-TB cases are not properly diagnosed; therefore, treatment in these cases mostly fails. A recent transmission model analysis suggests that most MDR-TB is transmitted rather than acquired [10].

In this regards, merely improving the treatment strategies is unlikely to greatly reduce the MDR-TB incidence [10]. Thus, using new technologies that would allow rapid detection of MDR-TB as well as implementing an improved treatment regimen could prevent further emergence of drug-resistant TB.

One of the key limitations of this study is the “referral bias” due to the studies included being done under the referral setting. Thus, it cannot fully represent the prevalence of MDR-TB in Tehran, Iran.

## Conclusions

There has been trend toward increasing MDR-TB rates, particularly in re-treatment. Hence, improved diagnosis and treatment strategies for MDR-TB should be highly prioritized.

## Notes

### Author contributions

Mohammad Javad Nasiri and Mohsen Heidary contributed equally.

### Competing interests

The authors declare that they have no competing interests.

## Figures and Tables

**Table 1:**
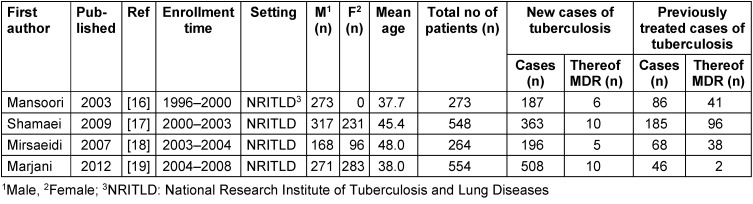
Characteristics of included studies

**Table 2:**
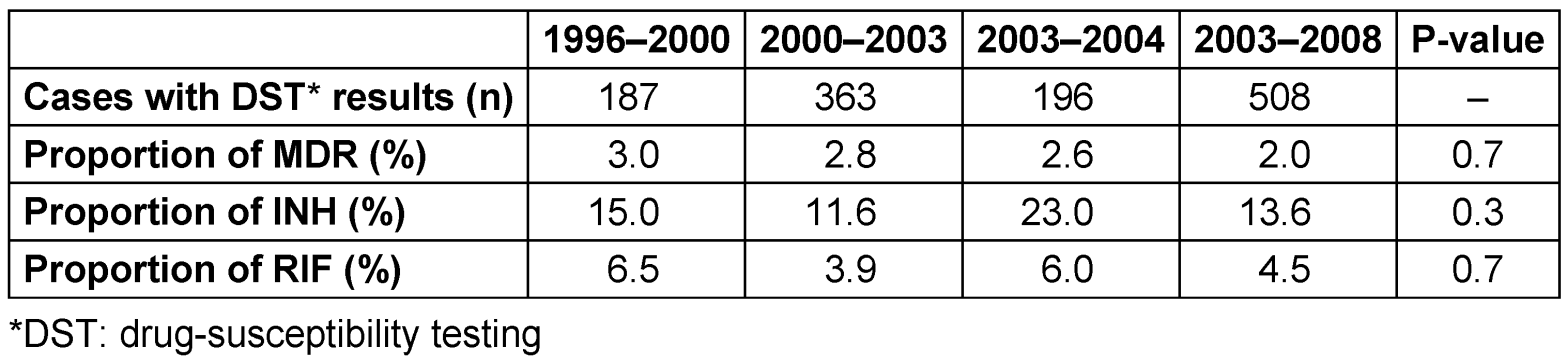
Trend of MDR-TB in new cases

**Table 3:**
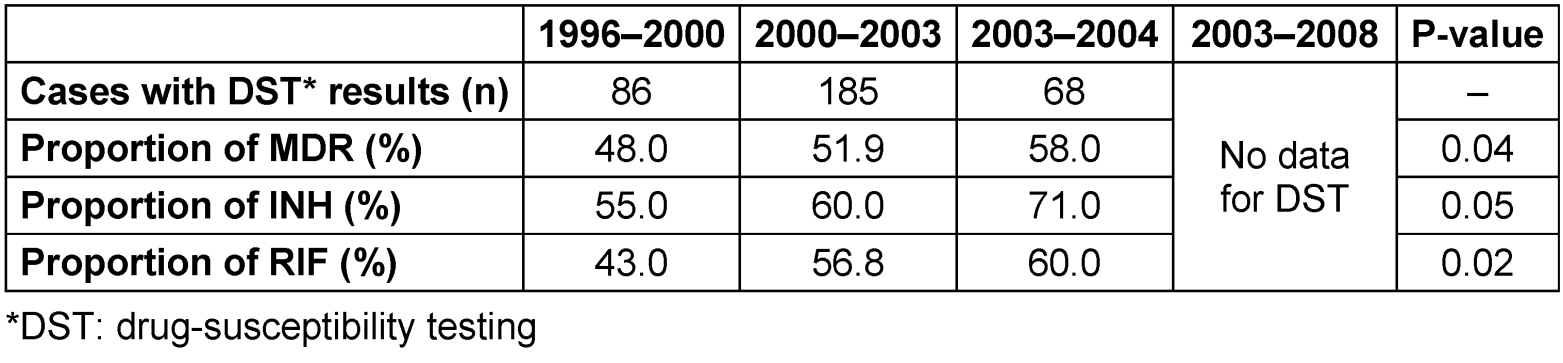
Trend of MDR-TB in previously treated cases
